# Elevated type I interferon-like activity in a subset of multiple sclerosis patients: molecular basis and clinical relevance

**DOI:** 10.1186/1742-2094-9-140

**Published:** 2012-06-22

**Authors:** Alexander Hundeshagen, Michael Hecker, Brigitte Katrin Paap, Charlotte Angerstein, Ole Kandulski, Christian Fatum, Christiane Hartmann, Dirk Koczan, Hans-Juergen Thiesen, Uwe Klaus Zettl

**Affiliations:** 1 Department of Neurology, Division of Neuroimmunology, University of Rostock, Gehlsheimer Str. 20, 18147, Rostock, Germany; 2 Institute of Immunology, University of Rostock, Schillingallee 68, 18057, Rostock, Germany

**Keywords:** Blood biomarker, IFN-beta, Multiple sclerosis, MX1, Pharmacological effects, Signaling pathway, Therapy response

## Abstract

**Background:**

A subset of patients with multiple sclerosis (MS) shows an increased endogenous IFN-like activity before initiation of IFN-beta treatment. The molecular basis of this phenomenon and its relevance to predict individual therapy outcomes are not yet fully understood. We studied the expression patterns of these patients, the prognostic value of an elevated IFN-like activity, and the gene regulatory effects of exogenously administered IFN-beta.

**Methods:**

Microarray gene expression profiling was performed for 61 MS patients using peripheral blood mononuclear cells obtained before and after 1 month of IFN-beta therapy. Expression levels of genes involved in pathways either inducing or being activated by IFN-beta were compared between patients with high (MX1_high_ cohort) and low (MX1_low_ cohort) endogenous IFN-like activity. Patients were followed for 5 years and relapses as well as progression on the expanded disability status scale (EDSS) were documented.

**Results:**

Before the start of therapy, 11 patients presented elevated mRNA levels of IFN-stimulated genes indicative of a relatively high endogenous IFN-like activity (MX1_high_). In these patients, pathogen receptors (for example, *TLR7*, *RIG-I* and *IFIH1*) and transcription factors were also expressed more strongly, which could be attributed to an overactivity of IFN-stimulated gene factor 3 (ISGF3, a complex formed by *STAT1*, *STAT2* and *IFN regulatory factor 9*). After 1 month of IFN-beta therapy, the expression of many pathway genes was significantly induced in MX1_low_ patients, but remained unaltered in MX1_high_ patients. During follow-up, relapse rate and changes in EDSS were comparable between both patient groups, with differences seen between different types of IFN-beta drug application.

**Conclusions:**

Therapeutic IFN-beta induces the transcription of several genes involved in IFN-related pathways. In a subgroup of MS patients, the expression of these genes is already increased before therapy initiation, possibly driven by an overexpression of ISGF3. Patients with high and low endogenous IFN-like activity showed similar clinical long-term courses of disease. Different results were obtained for different IFN-beta drug preparations, and this merits further investigation.

## Background

Multiple sclerosis (MS) is a chronic inflammatory disease causing neuronal demyelination and axonal decay in the central nervous system. The impaired nerve conduction results in progressive neurological dysfunctions, ultimately leading to severe impairment of the patient [1]. No distinct etiology has so far been determined, but autoimmune processes, genetic dispositions and environmental factors have been associated with the disease [1-5]. The individual course of MS is highly heterogeneous making predictions of long-term prognosis challenging. The majority of patients experience a relapsing-remitting type of MS (RR-MS) during the first years of disease with episodically flourishing neurological dysfunctions [6-8]. To date, no curative therapy is available. An immunomodulatory therapy with IFN-beta is a common first-line approach for controlling exacerbations and progression in RR-MS. Three drug formulations of IFN-beta are available: subcutaneous (sc) IFN-beta-1a and IFN-beta-1b, and intramuscular (im) IFN-beta-1a [9,10]. The molecular mechanisms of action of these drugs have been studied extensively over the past years [11,12]. IFN-beta therapy, however, proved only partially effective, with a subset of patients not sufficiently responding to therapy. This means that, despite treatment, further disease progression can be observed in terms of relapses, worsening on the expanded disability status scale (EDSS), or formation of new brain lesions [13].

A recent focus of research has been the search for molecular biomarkers that can assist in diagnosis as well as in monitoring of disease activity and therapeutic effects. In this context, biomarkers are sought to predict long-term disease progression and individual therapy response with the aim of reducing the delay of effective therapy and lowering socioeconomic costs on ineffective drug applications [14,15]. However, none of the potentially prognostic biomarker candidates have so far been established and implemented in clinical practice. More than a hundred genes have been proposed as blood biomarkers, but most could not be independently confirmed in a recent study [16]. One widely accepted explanation for IFN-beta therapy failure is the formation of neutralizing antibodies (NAb). This occurs after a few months of treatment in 7 % to 35 % of the patients, dependent on the drug formulation [17]. NAbs are antibodies against recombinant IFN-beta that diminish its ability to induce the transcription of interferon-stimulated genes (ISGs) and hence reduce the bioactivity and the long-term clinical effects of the drug [18,19]. Thus far, hundreds of ISGs have been identified. They show a strong co-regulation upon stimulation with type I IFNs (IFN-alpha and IFN-beta) [20,21]. The mRNA induction of ISGs by IFN-beta therapy (in the absence of NAbs) has been demonstrated to be relatively short-lasting, with many ISGs returning to pre-treatment levels within 4 days [22,23]. One of the most prominent ISGs is *myxovirus resistance protein 1* (*MX1* or *MxA*). The blood expression of *MX1* has been shown to reflect the immunoregulatory activity of IFN-beta, and hence *MX1* has been clinically implemented to measure the effects of IFN-beta administration on gene regulation and to detect the presence of NAbs [24,25].

While *MX1* is a useful biomarker during IFN-beta treatment, it has been debated whether already prior to treatment the expression of *MX1* or other ISGs allows the prediction of individual long-term clinical outcomes. It has been observed that a subgroup of therapy-naïve MS patients shows an elevated endogenous ISG expression and, thus, an increased type I IFN-like activity [26]. Based on this finding, van Baarsen *et al*. [27] suggested that the pre-treatment IFN signature in the peripheral blood could serve as a prognostic biomarker of clinical responses to IFN-beta therapy. Comabella *et al*. [28,29] later reported an overexpression of ISGs before the start of IFN-beta treatment in clinical non-responders compared to responders. They observed that, upon treatment, the expression of these genes remained unaltered in non-responders, but was strongly upregulated in responders. They attributed the elevated IFN signature to increased amounts of phosphorylated *STAT1* and *IFN-alpha/beta receptor 1* (*IFNAR1*) in monocytes. Moreover, higher baseline levels of IFN-beta itself were detected in non-responders by Axtell *et al*. [30] and this was later confirmed by Bustamante *et al*. [29]. On the other hand, Feng *et al*. [31] detected a lower expression of ISGs in patients with active MS and found that this was linked to a subnormal phosphorylation of the transcription factor (TF) *STAT1*. They concluded that a defect in type I IFN signaling may predict the course of disease and responses to therapy. Recently, van der Voort *et al*. [32] studied a group of 116 RR-MS patients and observed that high *MX1* mRNA levels in the blood were significantly associated with a longer time to a first new relapse. A potentially beneficial effect of an elevated endogenous type I IFN response was also reported by Hesse *et al*. [33], who described a positive correlation of *MX1* expression and *IL-10* expression, and a negative correlation of *IL-10* expression and disease activity on magnetic resonance imaging in untreated MS patients.

The results of these studies have been somewhat inconsistent, possibly due to differences in the type of specimen analyzed, measurement technology, treatment strategy, definition of disease progression, and data analysis. Hence, there is a need to independently validate whether the individual IFN signaling activity, reflected by the expression of *MX1* and other ISGs, can predict the individual course of disease. Moreover, the underlying molecular physiology of type I IFN-like activity and the effects of IFN-beta therapy on the IFN-beta-related pathways have so far not been elucidated in a comprehensive manner. A deeper insight into these effects may help to better understand the mechanisms of action of the drug and to disclose transcript-based disease heterogeneity.

In this work, we investigated the molecular basis of high endogenous IFN-like activity by studying the pathways involved in IFN regulation and signaling. Furthermore, we analyzed the gene regulatory effects of IFN-beta therapy and the expression differences between MS patients with low and high pre-treatment IFN-like activity. To evaluate the prognostic power of this activity on therapy success, we examined the disease progression over the long-term course of MS.

## Methods

### Interferon pathways

To unravel the molecular basis that accounts for individual differences in the endogenous IFN-like activity, we looked for genes involved either in the pathways regulating IFN-beta expression or in the pathways triggered by IFN-beta. We searched the PubMed database for review articles published within the last 5 years addressing the respective IFN-beta-related pathways. Eleven reviews were selected [34-44], and we extracted the genes that were redundantly mentioned in these publications together with their mutual interactions (Additional file 1). A network of the genes visualizing the different types of interactions (for example, binding, activation and inhibition) was constructed using the Cytoscape software version 2.8.1 (Cytoscape Consortium, San Diego, CA, USA, http://www.cytoscape.org).

### Experimental setup and microarray data

This study comprises 61 patients suffering from RR-MS diagnosed according to the McDonald criteria [45]. The patients were prescribed sc IFN-beta-1a (n = 12), sc IFN-beta-1b (n = 25) or im IFN-beta-1a (n = 24) treatment. Blood samples were drawn immediately before the start of therapy (baseline) and after 1 month prior to the next drug application. Peripheral blood mononuclear cells (PBMC) were separated from the blood samples by Ficoll gradient and total RNA was extracted using the RNeasy Mini Kit (Qiagen, Hilden, Germany). The RNA was processed, labeled and hybridized to Affymetrix HG-U133 A and B or Plus 2.0 oligonucleotide microarrays according to the manufacturer’s protocols. To calculate the gene expression levels, we used custom chip definition files (CDFs) and applied the MAS5.0 algorithm. The custom CDFs (version 2.1.0, http://www.xlab.unimo.it/GA_CDF/) [46] define probesets composed of probes matching only a single gene based on the GeneAnnot (version 1.9) and GeneCards (version 2.41) databases [47]. In this way, an accurate assignment of probesets and genes could be achieved. This gave us an expression data set of about 17,000 genes, corrected for variation between the microarrays by loess normalization. The full data are available in the GEO database [GEO:GSE19285, GEO:GSE24427, GEO:GSE33464]. Further details on the experiments and the data preprocessing can be found elsewhere [48,49]. The studies were approved by the University of Rostock’s ethics committee and carried out according to the Declaration of Helsinki. Written informed consent was obtained from all patients before the blood sampling.

### Statistical analysis of the gene expression data

Based on the baseline *MX1* transcript levels as a measure of endogenous type I IFN-like activity, the patients were divided into two groups. The detected *MX1* signal intensities varied from 875 to 11,588 (Additional file 2). We set a cut-off at 3,000, which was two standard errors above the mean. Patients with *MX1* levels below the cut-off were allocated to the MX1_low_ cohort and those with *MX1* levels above the cut-off were allocated to the MX1_high_ cohort. The mean expression levels of the genes involved in the IFN-beta-related pathways were then compared between the MX1_high_ group and the MX1_low_ group at baseline as well as after 1 month using the Wilcoxon rank-sum test. We further compared the expression levels at both time-points within each group using the Wilcoxon signed-rank test. Expression differences with *P*-values below 0.01 were considered statistically significant. For the gene expression comparisons, only data from patients who received IFN-beta sc were considered since, due to the different drug application schedules for IFN-beta sc (3 to 4 injections per week) and IFN-beta im (1 injection per week), the interval between two subsequent drug injections differs. Since the blood sampling was always performed before the next injection, this has a considerable influence on the measured mRNA levels [22,23]. Statistical analyses have been computed with PASW Statistics version 18.0.0 (SPSS Inc., Chicago, IL, USA) and the data were imported into Cytoscape to visualize differences and changes in gene expression.

### Validation of the microarray data by real-time PCR

Quantitative real-time reverse transcription PCR was performed to confirm the gene expression levels quantified by the microarrays. The microarray experiments were conducted between 2003 and 2007, and the real-time PCR experiments were performed in 2012. Meanwhile, the total RNA samples were stored at −25 °C. RNA quantity and quality was determined using a NanoDrop 1000 Spectrophotometer (Thermo Scientific, Wilmington, DE, USA) and a 2100 Bioanalyzer (Agilent Technologies, Palo Alto, CA, USA), respectively. For 44 of the 61 patients, sufficient amounts of PBMC RNA were available for both the baseline sample and the sample obtained 1 month after the start of treatment. A set of genes was re-measured by real-time PCR in the samples of these patients. From each sample, 0.2 μg total RNA was reverse transcribed using the High Capacity cDNA Reverse Transcription Kit (Applied Biosystems, Carlsbad, CA, USA). The obtained cDNA was then measured in 384-well TaqMan Arrays with pre-designed TaqMan assay reagents according to the manufacturer’s instructions with a 7900HT Sequence Detection System (Applied Biosystems). The two samples from each patient were always processed in one batch. Fluorescence was measured during each PCR cycle, and thresholds at which the signal is detected above background (Ct values) were computed automatically using the SDS 2.3 and RQ Manager 1.2 software (Applied Biosystems). For four patients, the real-time PCR measurement was replicated. For those, we calculated the mean of the Ct values. The Ct values were then utilized for the analysis of differential expression applying Wilcoxon tests as described for the microarray data. Spearman’s rank correlation coefficients and *P*-values were computed to assess whether the real-time PCR data correlate with the microarray data.

### Stability of MX1 mRNA expression

An elevated type I IFN-like activity may either be characteristic for some patients or may be only a temporary phenomenon at the time of blood sampling (for example, due to an undiagnosed viral infection). We therefore evaluated whether the natural *MX1* expression is stable over time using the Affymetrix microarray data by Karlovich *et al*. [GEO:GSE16028] [50]. The data provide whole blood gene expression levels of 22 healthy individuals measured at five different time-points spanning a period of 6 months. We pre-processed the data in the same manner as we pre-processed our own data (see section “experimental setup and microarray data”). Correlation of *MX1* levels at study onset and after 6 months was assessed by Spearman’s rho. We performed an analysis of variance (ANOVA) to explore the impact of age, gender, time-point and the individual subjects on the measured *MX1* transcript signals. The linear ANOVA model was fitted using the R package “car” with type II sums of squares and F-test statistic.

### Analysis of transcription factor binding sites

We examined the regulatory regions of all genes involved in the IFN-beta-related signaling pathways for putative DNA-binding sites for the transcription factor complex ISGF3 (IFN-stimulated gene factor 3, Transfac database sequence motif identifier V$ISRE_01). Predictions of evolutionarily conserved transcription factor binding sites (TFBS) provided by the tfbsConsSites track (hg18) of the UCSC database were used for this purpose. For each gene, the regulatory region was assumed to be 1,000 base pairs up- and downstream of the respective transcription start site, which was taken from the GeneCards database (version 2.41).

### Clinical data evaluation

All patients were monitored for the occurrence of relapses, and EDSS assessments were performed after regular intervals of 3 months. The course of disease was followed for 5 years, even if the patients terminated the treatment during follow-up.

To detect possible pre-treatment differences in the patient population between the MX1_high_ and the MX1_low_ groups, we compared age and EDSS at baseline, elapsed time between diagnosis of definite MS and study onset, as well as the number of relapses 1 year prior to the study, applying the two-sample two-tailed *t*-test assuming equal variances with significance level alpha = 0.05. The sex ratio and the number of patients per administered IFN-beta formulation were compared between the cohorts with Fisher’s exact test at the alpha = 0.05 level of significance. Furthermore, these patient parameters were checked for correlation with the baseline expression levels of the pathway genes by calculating the Pearson correlation coefficient. Because of multiple testing, only correlation *P*-values below 0.001 were considered statistically significant.

We investigated the clinical relevance of endogenous IFN-like activity on the long-term course of disease by evaluating the progression of EDSS and the number of relapses during a 5-year follow-up period after the start of IFN-beta therapy. Mean EDSS scores and the cumulative number of relapses have been compared between the two patient groups using the *t*-test with Welch correction. Additionally, we compared the relapse-free survival during the follow-up by constructing right-censored Kaplan-Meier curves with PASW Statistics. The relapse-free survival curves for the MX1_high_ patient group and the MX1_low_ patient group have been tested for significant deviation by computing the log-rank test and the Cox proportional hazard ratio.

## Results

### Interferon pathways

We identified 46 genes that have redundantly been described in the literature to play a role in the pathways inducing IFN-beta transcription and the pathway that is activated by IFN-beta [34-44]. We complemented the list of genes with 10 genes that have repeatedly been reported as ISGs, including *MX1* (Additional file 1). The genes, and their interactions within the IFN-beta induction pathways and the IFN-beta effector pathway, will be summarized briefly below.

The expression of IFN-beta is physiologically induced after recognition of viral, bacterial or degraded endogenous nucleic acids by cellular receptors. This is mediated by the activation of two main pathways. The endosomal Toll-like receptors (TLRs) *TLR7-8* and *-9* activate a *MyD88*-dependent pathway with the signal transducers *IRAK4IRAK1* and *TRAF6*. The latter leads via *MAP3K7* (*TAK1*) to the phosphorylation of the kinases *IKKMAPK8* (*JNK*) and *MAPK14* (*p38*), resulting in the activation of the transcription factors *NFkappaBJUN* and *ATF2*, respectively. The other main pathway is initiated by the endosomal *TLR3*, as well as by the cytoplasmic receptors *IFIH1* (*MDA5*), *DDX58* (*RIG-I*) and *ZBP1*. They induce the phosphorylation of the IFN regulatory factors (IRF) *IRF3* and *IRF7* via *TRAF3* and *TBK1*/*IKK-epsilon*. These IRFs then homodimerize or heterodimerize and translocate to the nucleus. The cell membrane-bound *TLR4* can initiate both the *MyD88* pathway and the *TRAF3* pathway. The activated TFs bind to the promoter region of IFN-beta (*IFNB1*) and initiate its transcription [51]. Several cross-bridges between the *MyD88* and the *TRAF3* pathway exist [34-38,40,41,43,44].

Endogenously produced IFN-beta and exogenously injected IFN-beta bind to the IFN-alpha/beta receptor. This receptor comprises the subunits *IFNAR1* and *IFNAR2* with the associated kinases *TYK2* and *JAK1*. Once activated, the IFNAR complex phosphorylates *STAT1* and *STAT2*, which heterotrimerize with *IRF9* to form the ISGF3 complex (JAK/STAT pathway). ISGF3 translocates to the nucleus and binds the interferon-stimulated response element (ISRE), a DNA motif that can be found in the regulatory region of many ISGs. Activated STATs also form homodimers and heterodimers acting as TFs independent of *IRF9*[35-40,42,43]. The activation of STATs in response to IFN-beta was shown to differ among leukocyte subsets, with *STAT1* being mainly activated in monocytes and CD8+ T cells [52].

There are several positive and negative feedback loops in the type I IFN-related pathways. Many receptors, signal transducers and TFs within the IFN-beta induction pathway are themselves induced by IFNs, thereby amplifying IFN responses, for example *IRF7DDX58* and *IFIH1*[37,39]. As a negative feedback, the JAK/STAT pathway stimulates the transcription of several suppressor of cytokine signaling (SOCS) genes, which act as inhibitors on TLRs and JAKs, and target the IFNAR complex for proteasomal degradation [35].

### Patient characteristics

The 61 RR-MS patients (42 females, 19 males) in our study were between 18 and 63 years of age at the start of treatment with IFN-beta. The time elapsed between diagnosis of MS and therapy initiation was less than 9 months for 53 patients and between 25 and 134 months for the remaining 8 patients. At the time of blood sampling, there was no evidence of an ongoing infection based on clinical signs and laboratory testing. Over the complete 5-year observation period, 55 of the 61 patients have been followed at our clinic and 41 patients continuously received the prescribed IFN-beta therapy. Eleven of the 61 patients had relatively elevated baseline *MX1* expression levels. They were allocated to the MX1_high_ cohort, while the remaining 50 patients were allocated to the MX1_low_ cohort. There was no significant difference between the two patient groups in terms of age, sex ratio and baseline EDSS. The time between diagnosis and study onset, as well as the number of relapses during the year prior to the study, were comparable (Table 1). No significant correlation between any of these patient characteristics and the pre-treatment expression levels of the 56 pathway genes could be observed.

**Table 1:** Clinical and demographic parameters of the two patient cohorts

**Demographic and clinical characteristics**	**MX1**_**high**_**(n = 11)**	**MX1**_**low**_**(n = 50)**	***P* - value**
Age at study onset (years)	34.8 ± 8.7	38.3 ± 10.4	0.312
Time from diagnosis to therapy (months)	2.4 ± 2.4	11.6 ± 26.6	0.258
EDSS at baseline	2.0 ± 1.5	1.7 ± 1.1	0.510
Number of relapses during the year prior to study onset	1.2 ± 0.4	0.9 ± 0.6	0.172
Gender (women/men)	8/3	34/16	1.000
Type of IFN-beta administered (1a im/1a sc/1b sc)	4/3/4	20/9/21	0.826

Mean ± standard deviation are given for age, disease duration and expanded disability status scale (EDSS) at study onset as well as for the number of relapses in the year prior to treatment. These parameters were tested for differences between patients with low and high endogenous IFN-like activity (patient groups MX1_low_ and MX1_high_) applying the *t*-test. For the sex ratio and the administered IFN-beta products, the absolute patient numbers are given and *P*-values as calculated by Fisher’s exact test. im, intramuscular; sc, subcutaneous.

### Expression levels and dynamics dependent on MX1 status

**Figure 1:**
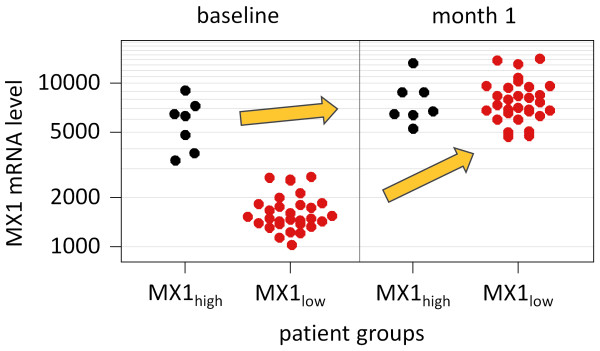
***MX1*expression levels immediately before and 1 month after the start of therapy with subcutaneous IFN-beta.** The measured signal intensities are presented in log scale due to the skewed data distribution. Patients with elevated *MX1* transcript levels at baseline (MX1_high_, n = 7, black dots) showed only a weak gene induction after 1 month of treatment (*P*-value = 0.156). In contrast, individuals in the MX1_low_ group (n = 30, red dots) had a significant increase in expression up to the levels of the MX1_high_ group (*P*-value = 1.9 × 10^-9^). The figure was drawn using the function “ehplot” of the R package “plotrix”.

For comparison of the PBMC gene expression between the two patient groups and between the two time-points, only patients who received IFN-beta sc were considered (see Methods). Of these individuals, 7 patients were in the MX1_high_ group and 30 patients were in the MX1_low_ group. After 1 month of therapy, the *MX1* expression levels of the MX1_high_ cohort remained relatively unchanged whereas MX1_low_ patients showed a significant induction up to the levels of the MX1_high_ group (Figure 1). Similar expression patterns were observed for other ISGs.

**Figure 2:**
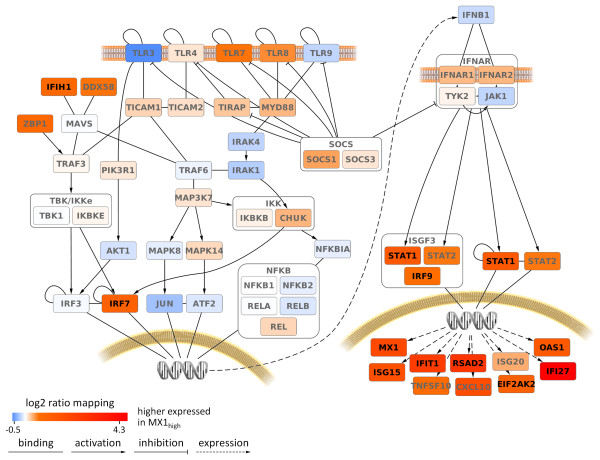
**Pre-treatment expression of TLR, RIG-I and IFN-beta pathway genes compared between the two patient groups.** The pathways that induce type I IFN expression via *TRAF3* and *MyD88* are shown on the left and the pathway that is triggered by IFN-beta (JAK/STAT pathway) is shown on the right. Each gene is colored by the magnitude of mean expression difference between the MX1_high_ group (n = 7) and the MX1_low_ group (n = 30). Black node labels indicate significant differences (*P*-value < 0.01). Aside from *MX1* as an established biomarker of IFN activity, other ISGs also showed higher levels of expression in the MX1_high_ patient cohort before initiation of subcutaneous IFN-beta therapy. The elevated endogenous IFN-like activity of these patients is likely the result of elevated amounts of the transcription factors *STAT1*, *STAT2* and *IRF9*, which form the ISGF3 complex. DNA-binding sites for this complex can be found in the promoters of RIG-I-like helicases (*IFIH1*, *DDX58*), suppressor of cytokine signaling (SOCS) genes and the transcription factor *IRF7*, which were also expressed at higher levels in MX1_high_ patients (see Additional file 3). Other genes such as *IRF3*, the NFkappaB members, *IFNB1* and the IFN-alpha/beta receptors were not differentially expressed.

When comparing the mean baseline expression of the 56 pathway genes between both cohorts, 11 genes were expressed in the MX1_high_ group at a significantly higher level. Elevated transcript levels were found for cytoplasmic receptors (for example, *IFIH1*), TFs (for example, *IRF7*, *IRF9* and *STAT1*) and, as expected, most ISGs (Figure 2 and Additional file 3). Other receptors, such as *TLR7* and *TLR8*, as well as the transcription factor *STAT2*, also showed elevated expression in MX1_high_ patients, but with *P*-values > 0.01. We found no significant difference in expression at the mRNA level for natural IFN-beta (*IFNB1*) and its receptors *IFNAR1* and *IFNAR2*.

**Figure 3:**
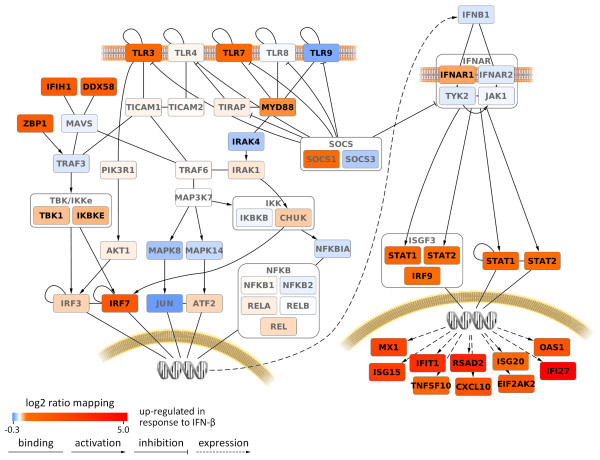
**IFN-beta-related pathways and gene expression changes in MX1**_**low**_**patients during the first month of IFN-beta therapy.** The log-2 ratios of the mean gene expression levels before and after 1 month of subcutaneous IFN-beta therapy are shown by the color of the gene nodes. Genes with significant expression changes are labeled in black (*P* < 0.01). Receptors, transcription factors and ISGs that were elevated in the MX1_high_ group (n = 7) at baseline (Figure 2) were strongly upregulated under therapy in MX1_low_ patients (n = 30), whereas *TLR9* and *IRAK4* were significantly downregulated in this patient group.

During the first month of IFN-beta sc therapy, the MX1_high_ group showed no significant change in the expression of the IFN-beta-related pathway genes (Additional file 4). In contrast, 25 of the 56 genes were found to be significantly modulated in MX1_low_ patients, with 23 genes being upregulated and two genes (*TLR9* and *IRAK4*) being downregulated in response to therapy (Figure 3). The upregulated genes comprised all the 11 genes that were significantly elevated in the MX1_high_ patient group at baseline. After 1 month of therapy, there was no difference in expression of the genes between the two patient groups (Additional file 5).

**Figure 4:**
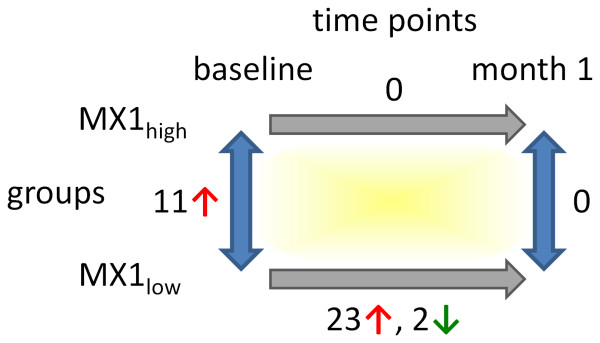
**Summary of the gene regulatory effects of IFN-beta therapy and the differences in expression between the two patient cohorts.** Before the start of therapy, 11 genes were expressed at significantly higher levels in the group of patients with elevated IFN-like activity (MX1_high_, n = 7). The pathway expression profile of those patients was not significantly influenced by subcutaneous IFN-beta therapy. In comparison, 25 genes were up- or downregulated in response to therapy in the MX1_low_ group (n = 30), including all 11 genes that were elevated in the MX1_high_ group at baseline. After 1 month of therapy, no significant differences in gene expression could be observed between the cohorts.

The differences in gene expression over time and between the cohorts are summarized in Figure 4 and in Additional file 3. The pathway visualizations are available from the authors as Cytoscape session files upon request.

### Real-time PCR analysis of differential gene expression

Of the 56 pathway genes, 14 were re-measured by real-time PCR. Overall, the PCR analyses confirmed the microarray results. Despite the long storage of the samples, the mRNA levels obtained by the two techniques correlated significantly for all genes except *TLR9* (Additional file 2). Of the 11 MX1_high_ patients, nine were contained in the PCR data. As in the microarray data, they also showed a higher pre-treatment expression of *MX1* (Ct values < 26.7) than all MX1_low_ patients in the PCR data. In the subgroup of patients treated with IFN-beta sc, there were five MX1_high_ patients and 21 MX1_low_ patients. Although sample sizes were smaller, the expression differences observed in the Affymetrix microarray data were also seen in the real-time PCR data: at baseline, *MX1*, *MYD88*, *RSAD2*, *STAT1* and *STAT2* were expressed at higher levels in MX1_high_ patients, and they were significantly upregulated during therapy only in the MX1_low_ cohort (Additional file 3).

### ISGF3 is a central regulator of type I IFN signaling

Predicted TFBS for ISGF3 were found in the promoter regions of 10 out of the 56 pathway genes (Additional file 3). There was a marked overlap when comparing the genes putatively regulated by the ISGF3 complex with those genes overexpressed in MX1_high_ patients at baseline. The receptors *IFIH1*, *DDX58* and *ZBP1*, two ISGs (*CXCL10*, *ISG20*), *IFNB1*, the pathway inhibitors *SOCS1* and *SOCS3*, as well as the TFs *IRF7* and *STAT2* contained predicted DNA-binding sites for ISGF3 in their promoter region. In this way, ISGF3 forms multiple positive and negative regulatory feedback loops. We conclude from this analysis that a higher expression and activity of the ISGF3 complex is most likely responsible for the higher endogenous type I IFN-like activity seen in a subset of patients.

### Stability of MX1 mRNA expression

In the time-course expression data by Karlovich *et al*. [50], we observed that approximately one-fifth of the healthy subjects showed relatively elevated *MX1* transcript levels in the blood, which is very similar to the proportion we ascertained for the MS patients in our study. Differential endogenous type I IFN-like activities therefore seem to be not exclusive for MS patients, and may rather demonstrate individual variation of innate immunity. Moreover, high *MX1* expression at study onset correlated strongly with the levels measured after 6 months (*P* = 0.00003). This revealed that the natural course of *MX1*, and thus the IFN-beta-like activity, is stable over time. ANOVA confirmed this result; the variance in the data could be explained best by intersubject differences (*P* = 3.6 × 10^-12^), while the effects of age and gender were not significant. The *MX1* expression data of the 22 healthy individuals are shown in Additional file 6.

### MX1 status and clinical outcome

**Figure 5:**
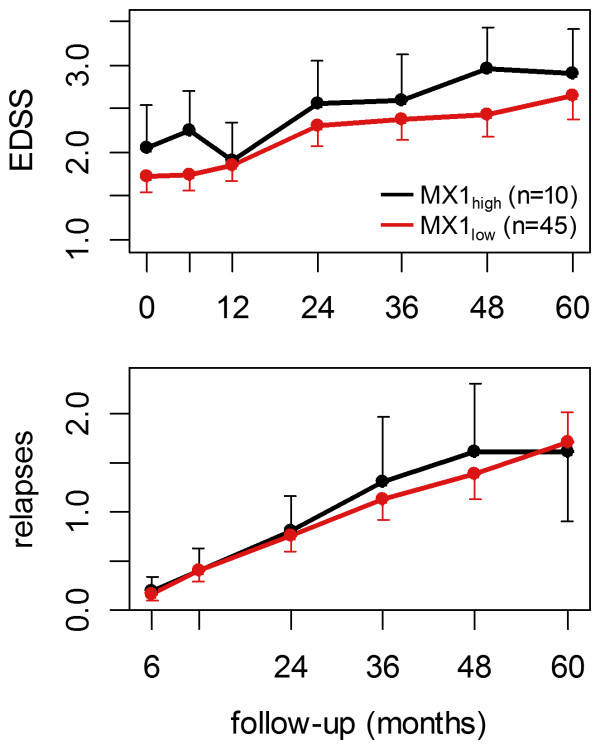
**Long-term progression of disability and cumulative number of relapses.** Mean time-courses are shown in black for the MX1_high_ group and in red for the MX1_low_ group. The change of disability according to the expanded disability status scale (EDSS) as well as the relapse rate during the 5-year follow-up period were similar for both cohorts (Welch’s *t*-test *P*-values > 0.2). MX1_high_ patients presented on average a slightly worse EDSS at baseline, and this nonsignificant difference persisted during the treatment. Error bars represent standard errors.

**Figure 6:**
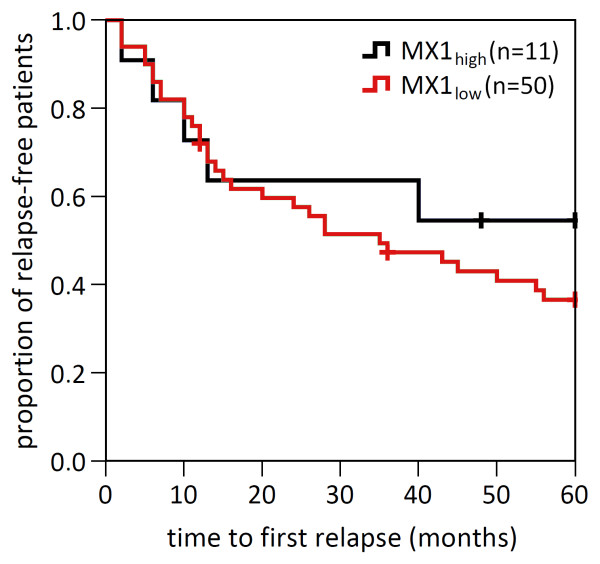
**Kaplan-Meier survival curves for patients with low and high endogenous IFN-like activity.** The Kaplan-Meier curves show the proportion of relapse-free patients in the MX1_high_ group (black curve) and in the MX1_low_ group (red curve). Vertical dashes indicate right-censored patients that dropped out of clinical surveillance (n = 3). In particular, during the first 18 months after therapy initiation, the proportions of relapse-free patients declined in a similar manner for both cohorts. Overall, the difference between both patient groups was not significant according to the log-rank test (*P* = 0.424) and the hazard ratio (0.684, confidence interval: 0.266 to 1.761).

To evaluate whether the endogenous IFN-beta-like activity is associated with individual disease progression and IFN-beta therapy outcome, we compared the MX1_high_ group and the MX1_low_ group in terms of EDSS and relapses as clinical measures of the long-term course of disease. The EDSS-defined changes of disability differed considerably between the individual patients. However, when comparing the mean EDSS of the two patient groups, the course of progression was very similar (Figure 5). After 5 years, the EDSS of the MX1_high_ cohort and the MX1_low_ cohort had risen, on average, by 0.85 and 0.92 points, respectively. The mean 5-year relapse rate also did not differ significantly between the MX1_high_ group (1.6) and the MX1_low_ group (1.7) (Figure 5). Accordingly, the Kaplan-Meier curves for relapse-free survival of both groups were very similar in their decline, especially during the first 2 years (Figure 6). For the complete 5-year follow-up period the calculated log-rank statistic (*P* = 0.424) and the hazard ratio (0.68, confidence interval: 0.27 to 1.76) showed no significant difference in the proportion of relapse-free patients between the cohorts.

However, when comparing the relapse rate between MX1_high_ patients and MX1_low_ patients separately for the three different drug formulations, we could observe significant differences (Additional files 7 and 8). In the subgroup of patients treated with IFN-beta-1a im, all the MX1_high_ patients (n = 4) were free of relapses over 5 years whilst the MX1_low_ patients (n = 17) experienced 1.6 relapses on average. In contrast, within the patient group receiving IFN-beta-1b sc, the MX1_high_ cohort (n = 3) had a much higher average number of relapses than the MX1_low_ cohort (n = 20) (2.7 versus 1.3). MX1_high_ patients and MX1_low_ patients had similar changes on the EDSS for each of the drug preparations. However, patients receiving IFN-beta im were diagnosed and treated at an earlier stage of the disease (mean baseline EDSS: 1.0) than those receiving IFN-beta sc (mean baseline EDSS: 2.3), and they were also more stable during the follow-up period.

## Discussion

Our results revealed that approximately one-fifth of MS patients show an elevated endogenous type I IFN-like activity prior to IFN-beta treatment and that this phenomenon can be explained by a higher expression and activity of the ISGF3 complex. This complex comprises the TFs *STAT1STAT2* and *IRF9*, and is not just a key regulator of ISGs, but is also important to induce diverse genes involved in IFN regulation and signaling [37,53]. Therefore, patients with high levels of the IFN activity biomarker *MX1* (MX1_high_ cohort) also showed high levels of other ISGs and several pathway genes, which in turn can control type I IFN transcription, forming positive feedback loops [39]. The link between ISGF3 and IFN-like expression signature is supported by the results of the TFBS analysis, which revealed that the list of ISGF3 target genes to a certain extent resembles the list of genes elevated in MX1_high_ patients. Our study is limited by the fact that we measured the gene expression in PBMCs at the mRNA level, which does not necessarily correlate with the amounts of active proteins due to post-transcriptional gene regulation, for example by microRNAs [54,55]. However, earlier studies at the protein level have already shown that the phosphorylation status of the ISGF3 component *STAT1* correlates with the ISG expression in MS patients [31].

We observed increased pre-treatment levels of the single-stranded RNA receptors *TLR7* and *TLR8* in MX1_high_ patients compared with MX1_low_ patients. This may also be mutually linked to an overactive JAK/STAT pathway in the MX1_high_ group of patients. The expression differences for these two TLR receptors did not reach the significance threshold (*P*-values < 0.01). However, this may be due to the fact that a different Affymetrix chip type (U133 Plus 2.0) was used for the samples of patients treated with IFN-beta-1a sc than for the others (U133 A and B). For some genes, including these two TLRs, differences in the custom probesets between both chip types led to increased variation in the data and thus somewhat higher *P*-values (Additional files 2 and 3).

Similar to our patients, we also found a relatively high type I IFN-like activity in a subset of healthy individuals, and *MX1* levels in the respective individuals remained elevated over the whole study period of 6 months. Therefore, independent of MS disease, some people seem to have a constitutive, robust signature of immune activation, while others do not. It has been described that the immune defense program is, in general, slightly more active in MS [26,56]. However, we could not evaluate this aspect, since we used external data on the whole blood of healthy subjects where it is difficult to compare the absolute mRNA signals with our PBMC data on MS patients. Still, the data on the healthy subjects provided strong evidence that the observed increased IFN-beta-like activities are not just the result of short-term hidden viral infections. This is further supported by our finding that the IFN-beta gene itself (*IFNB1*) was not expressed at higher levels in the MX1_high_ group as would be expected during an activated antiviral response (hence we used the term “IFN-like activity”). However, *IFNB1* transcript levels were virtually undetectable in our Affymetrix microarray data (Additional file 2), and we have not measured them with real-time PCR. Others have used more sensitive methods and could detect an elevated endogenous IFN-beta expression in PBMCs at baseline in IFN-beta therapy non-responders [29], which they believed to be causal for an elevated type I IFN signature [28]. We could not confirm these conclusions with the data from our study.

During IFN-beta sc therapy, patients with relatively high ISG expression at baseline showed no significant modulation in the expression of the genes involved in IFN-beta-related pathways. In contrast, patients in the MX1_low_ group showed a strong gene induction after 1 month of treatment (Figure 4). The congruency between the set of genes primarily elevated in MX1_high_ patients and the genes induced by therapeutic IFN-beta in MX1_low_ patients was striking. Possibly, higher levels of *MX1* and its co-expressed genes might be observed during therapy in both patient groups, if the blood expression is measured in the first 12 h after an IFN-beta injection [22,23]. Moreover, since we only measured the changes in mRNA expression, additional experiments on the protein level are needed to further investigate specific issues, for example the correlation between the amounts of STAT transcripts and the amounts of phosphorylated STAT proteins. Similarly, while we observed no significant blood expression differences for IFN-alpha/beta receptors, we can only speculate about the amounts and cell type-specific localization of the corresponding proteins. Comabella *et al*. [28,29] used flow cytometry to measure the expression of *IFNAR1* on monocytes. In patients with ongoing disease activity despite IFN-beta treatment, they found a higher baseline expression of this receptor [28,29]. Dupont *et al*. [57] showed, using *in vitro* experiments with Jurkat cells, that the surface expression of *IFNAR1* decreases after IFN-beta application and that the cells were subsequently unresponsive to further IFN-beta treatment for some hours. We may miss such findings in the PBMC population. Additionally, receptor polymorphisms, or alternatively spliced transcripts of these receptors, could play a role. Different splice variants of the *IFNAR2* receptor show different biologic responses to endogenous and exogenous IFN-beta and there is evidence that IFN-beta immunogenicity may be, in part, related to *IFNAR2* isoform expression [58]. However, since NAbs were not routinely measured for our patients, we were not able to investigate this possible association of IFNAR expression and NAbs.

A further interesting observation was that the levels of *TLR3* and *-7* were significantly upregulated, while *TLR9* was significantly downregulated, in MX1_low_ patients during therapy. Pre-treatment levels of *TLR3* were somewhat lower for MX1_high_ patients (*P* = 0.059), but the expression also strongly increased within 1 month of IFN-beta administration in this patient group (*P* = 0.016). *TLR7* is engaged in the maturation of dendritic cells, which play a role in bridging innate and adaptive immunity [59]. Upregulation of *TLR7* on dendritic cells by IFN-beta has been shown to inhibit the differentiation of Th17 cells [60], which have been recognized as proinflammatory lymphocytes involved in MS pathogenesis [61]. TLR-mediated signaling and IFN signaling is counter-regulated by *SOCS1*, the mRNA levels of which were upregulated in the MX1_low_ patient group during therapy (*P* = 0.017). A genetic risk factor for MS has been identified in the *SOCS1* promoter region recently [62].

We addressed the clinical importance of endogenous IFN-like activity and its prognostic power to predict disease progression and clinical response to IFN-beta therapy, an issue that has been debated with contradictory results [27-33]. Some studies suggest that baseline overexpression of ISGs may indicate pre-activated pathways in a way that IFN injections are less effective, which may explain suboptimal therapeutic responses [28,30]. However, a correlation between an elevated endogenous type I IFN signature and a worse course of disease could not be confirmed in our study, even though the MX1_high_ patient group showed a much weaker biologic response within the first treatment month, at least regarding the mRNA levels of the 56 examined pathway genes. Over the 5-year follow-up period, neither the average number of relapses nor the increase in EDSS were significantly different between patients with low and high baseline *MX1* levels. On the contrary, in a study by van der Voort *et al*. [32], high *MX1* mRNA expression was predictive of a longer time to a first new relapse. However, less than half of their patients that were initially therapy-naive started an immunomodulatory treatment during follow-up, and the baseline *MX1* levels did not differ significantly between responder status groups for the subset of treated patients. The beneficial effect of an increased IFN-like activity thus may be observed primarily in untreated patients. This is supported by the results of Hesse *et al*. [33], who found an indirect negative correlation between the number of active lesions and the blood expression of *MX1* in untreated patients. We hypothesize that a higher endogenous IFN-like activity does not imply IFN-beta therapy non-response, but might be positive in untreated patients. Nevertheless, the question remains whether MX1_high_ patients could benefit more from other treatments than from IFN-beta.

Our analysis revealed some differences in the relapse rates of the MX1_high_ group and the MX1_low_ group when analyzing the data for each IFN-beta drug preparation separately. However, this result must be interpreted with caution for two reasons: firstly, for each of the three therapy regimes, the number of patients per MX1 group is relatively small; and secondly, we did not systematically test for NAbs after long-term treatment, but their appearance might have led to reduced therapeutic benefits in some of the patients. In our data, MX1_high_ patients treated with once weekly 30 μg IFN-beta-1a im experienced no relapse during the 5-year follow-up period, while MX1_high_ patients receiving high-frequent high-dose IFN-beta-1b sc experienced, on average, 2.7 relapses. It is tempting to speculate about the reasons for this discrepancy. One possible explanation might be that only intermediate levels of IFN-beta are beneficial. Patients with low inherent type I IFN-like activity might need frequent IFN-beta therapy, whereas in MX1_high_ patients such a more intense IFN administration may not yield the desired effects, and possibly raises the chance of NAb development. An alternative explanation might be found in the disease duration. Patients treated with IFN-beta-1a im were, according to the EDSS at study onset, in an earlier stage of MS, and it is thus conceivable that a higher endogenous IFN signature combined with IFN-beta treatment might be preferentially favorable early in disease. Therapy-specific studies with larger cohorts are needed to test whether *MX1* has the potential to support the choice of IFN-beta drug preparation for the individual patient. Such further investigations must carefully consider the issues of dosage and frequency of IFN-beta administration, and should take the issue of NAbs into account.

## Conclusions

In conclusion, an elevated type I IFN-like activity was observed in the blood of a subset of MS patients as well as a subset of healthy individuals. We showed for the healthy subjects that this gene expression signature is robust over time and is therefore probably not the result of a transient latent infection. For the MS patients we demonstrated that the expression of genes involved in IFN-related pathways is strongly induced by IFN-beta therapy in those patients with low endogenous IFN-like activity, but is only weakly induced in patients with high endogenous IFN-like activity. The baseline gene expression pattern characteristic for the latter group resembles the pattern induced by therapeutic IFN-beta. While the natural IFN-beta and its receptors were expressed at similar mRNA levels in both groups, genes of the ISGF3 transcription factor complex were expressed significantly more in patients with an elevated endogenous IFN-beta-like signature. Our TFBS analysis led us to the hypothesis that the activity of the ISGF3 complex is crucial for the individually different activation status of the IFN pathways. Our study, which was limited by a relatively small sample size, did not confirm that the IFN signature prior to treatment with IFN-beta is predictive of the individual long-term course of MS, but different outcomes were observed for different drug formulations. This deserves further investigation with more sensitive and cell type-specific analyses on the RNA and protein level.

## Abbreviations

ANOVA, Analysis of variance; CDF, Chip definition file; Ct, Cycle threshold; EDSS, Expanded disability status scale; IFN, Interferon; IFNAR, IFN-alpha/beta receptor; IL, Interleukin; im, Intramuscular; IRF, IFN regulatory factor; ISG, Interferon-stimulated gene; ISGF3, IFN-stimulated gene factor 3; ISRE, Interferon-stimulated response element; MS, Multiple sclerosis; MX1, Myxovirus resistance protein 1; NAb, Neutralizing antibody; PBMC, Peripheral blood mononuclear cells; PCR, Polymerase chain reaction; RR-MS, Relapsing-remitting multiple sclerosis; sc, Subcutaneous; SOCS, Suppressor of cytokine signaling; STAT, Signal transducer and activator of transcription; TFBS, Transcription factor binding sites; TF, Transcription factor; TLR, Toll-like receptor.

## Competing interest

UKZ received research support as well as speaking fees from Bayer, Biogen Idec, Merck Serono, Novartis, Sanofi-Aventis, and Teva. MH received speaking fees from Bayer HealthCare. The other authors declare that they have no competing interests.

## Authors’ contributions

UKZ and HJT inspired and directed the work. OK, CF, CH and UKZ were responsible for patient care and clinical documentation. The lab experiments were performed by BKP and DK. AH and MH carried out the analysis and interpretation of the data, drafted the paper and prepared all tables and figures. CA, BKP and DK assisted in compiling the literature as well as in interpreting the results and contributed to the writing of the manuscript. The paper was corrected, improved and completed by HJT and UKZ, and all authors read and approved the final version.

## Supplementary Material

Additional file 1 References to review articles addressing the genes involved in IFN-related pathways. Click here for file

Additional file 2 **This table provides the preprocessed microarray data of the 56 pathway genes for all 61 patients before therapy initiation (baseline) and after 1 month of IFN-beta treatment.** Additionally, it contains the real-time PCR data for 14 of these genes that were obtained for 44 of the patients. Click here for file

Additional file 3 **Genes involved in the pathways regulating IFN-beta expression and in the pathway that is activated by IFN-beta.** Mean microarray signal intensities, calculated Wilcoxon test *P*-values as well as ISGF3 DNA-binding site information are provided for all genes. Moreover, for 14 of the genes, the results of the real-time PCR analysis are also shown. Click here for file

Additional file 4 **IFN-beta-related signaling pathways displaying the pharmacological effects on gene expression for the MX1**_**high**_**patient group after 1 month of treatment with IFN-beta sc.** Click here for file

Additional file 5 Expression of TLR, RIG-I and IFN-beta pathway genes compared between both cohorts at 1 month after start of IFN-beta sc therapy. Click here for file

Additional file 6 **The natural course of*MX1*mRNA expression is shown for 22 healthy individuals over a period of 6 months.** Click here for file

Additional file 7 Comparison between both cohorts in terms of EDSS changes and cumulative number of relapses during a 5-year observation period displayed separately for each IFN-beta drug preparation. Click here for file

Additional file 8 The proportion of relapse-free patients during 5 years of follow-up for both patient groups, dependent on the administered IFN-beta drug preparation. Click here for file
