# Supplementary material for: Elevated type I interferon-like activity in a subset of multiple sclerosis patients: molecular basis and clinical relevance
Source: J Neuroinflammation. 2012 Jun 22;9:140. doi: 10.1186/1742-2094-9-140 (PMC3464734; doi:10.1186/1742-2094-9-140)
Supplement: Additional file 5 — Expression of TLR, RIG-I and IFN-beta pathway genes compared between both cohorts at 1 month after start of IFN-beta sc therapy. [file 1742-2094-9-140-S5.pdf]

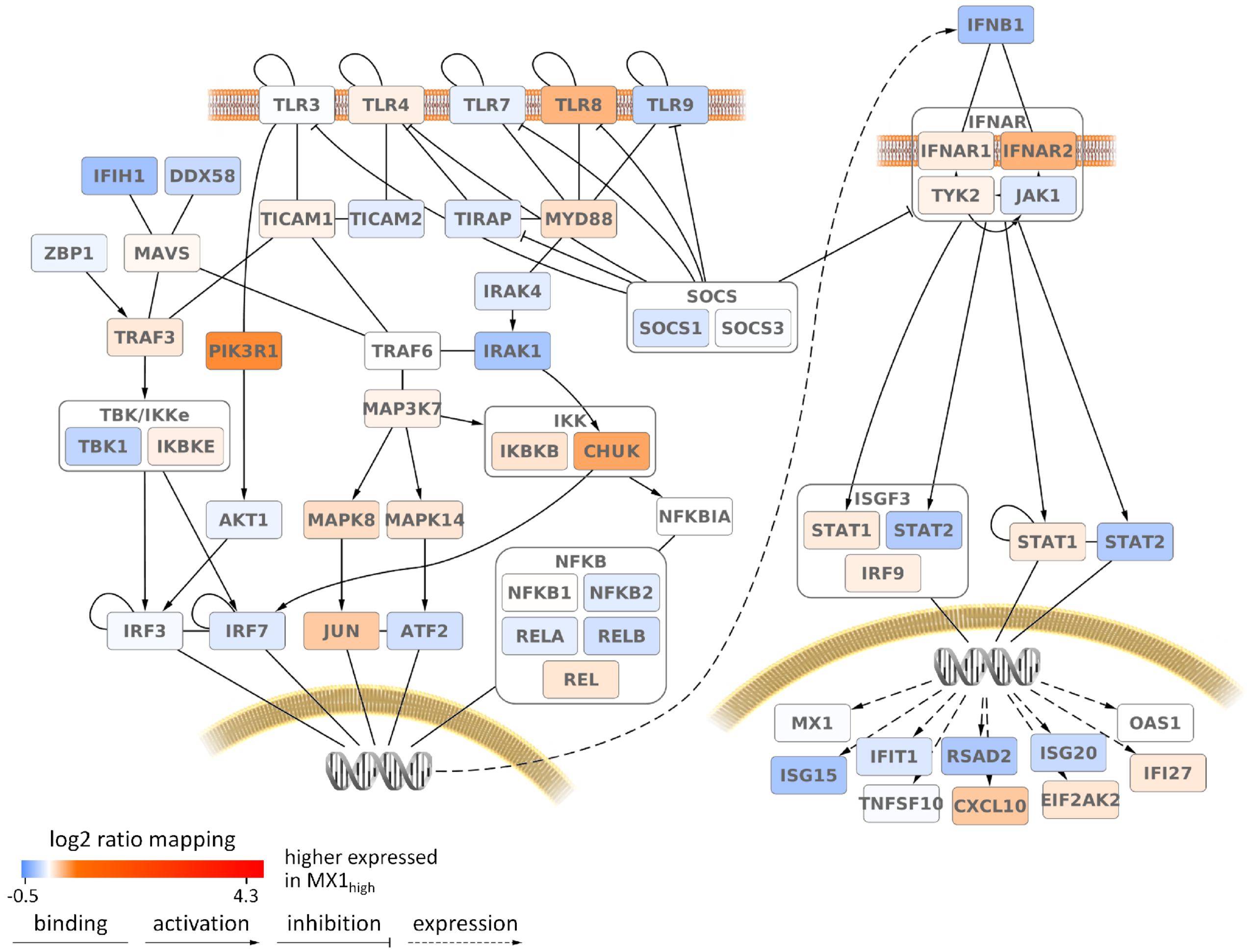

Additional file 5: Differences in the expression of type I IFN pathway genes between the $MX1_{high}$ patient group (n=7) and the $MX1_{low}$ patient group (n=30) one month after start of IFN-beta sc. therapy.

Magnitudes of mean expression differences are visualized by the color of each gene. While 11 genes were significantly higher expressed in the $MX1_{high}$ group at baseline (Figure 2, main manuscript), the transcript levels of the genes were comparable between the two groups during the therapy (p-values >0.01).
