# Supplementary material for: Elevated type I interferon-like activity in a subset of multiple sclerosis patients: molecular basis and clinical relevance
Source: J Neuroinflammation. 2012 Jun 22;9:140. doi: 10.1186/1742-2094-9-140 (PMC3464734; doi:10.1186/1742-2094-9-140)
Supplement: Additional file 6 — The natural course ofMX1mRNA expression is shown for 22 healthy individuals over a period of 6 months. [file 1742-2094-9-140-S6.pdf]

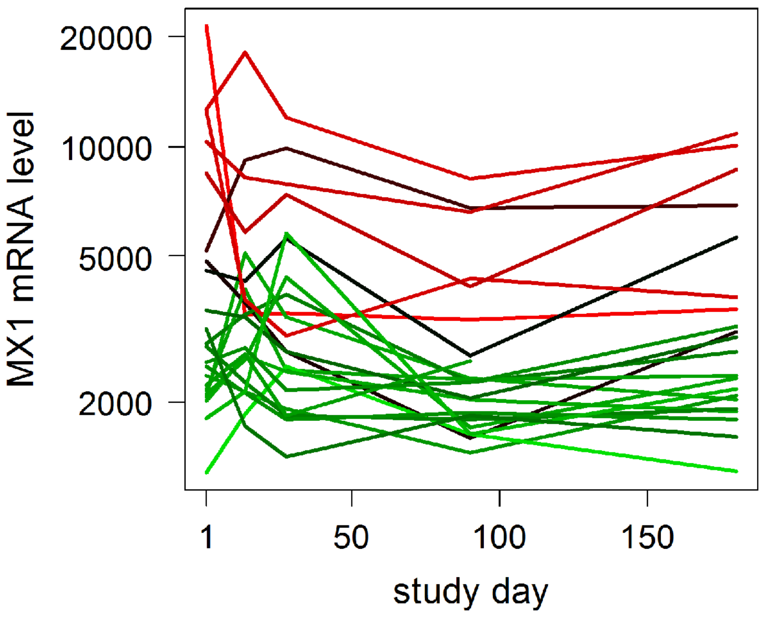

Additional file 6: MX1 time-course in healthy individuals.

Microarray data by Karlovich et al. (Genomics, 2009, GSE16028) were used to evaluate the natural course of MX1 expression. Transcript levels of MX1 were measured in whole blood samples of 22 healthy individuals at five time-points spanning an interval of six months. Two of these patients experienced a severe adverse event during the study (for more information the reader is referred to Karlovich et al., Genomics, 2009). Like in our expression data on MS patients, the distribution of MX1 expression levels is skewed. At study onset 5 subjects had relatively high MX1 levels (red lines), 3 subjects had intermediate levels in the signal range of 4000 to 6000 (black) and 14 subjects had low MX1 levels (green). Individuals with elevated MX1 expression at study onset also presented high levels after six months (Spearman's rho=0.79; p-value=0.00003). This suggests that an elevated type I IFN-like activity not just resembles a short-term hidden anti-viral response, but rather characterizes the subjects over the long term.
