# Supplementary material for: Elevated type I interferon-like activity in a subset of multiple sclerosis patients: molecular basis and clinical relevance
Source: J Neuroinflammation. 2012 Jun 22;9:140. doi: 10.1186/1742-2094-9-140 (PMC3464734; doi:10.1186/1742-2094-9-140)
Supplement: Additional file 7 — Comparison between both cohorts in terms of EDSS changes and cumulative number of relapses during a 5-year observation period displayed separately for each IFN-beta drug preparation. [file 1742-2094-9-140-S7.pdf]

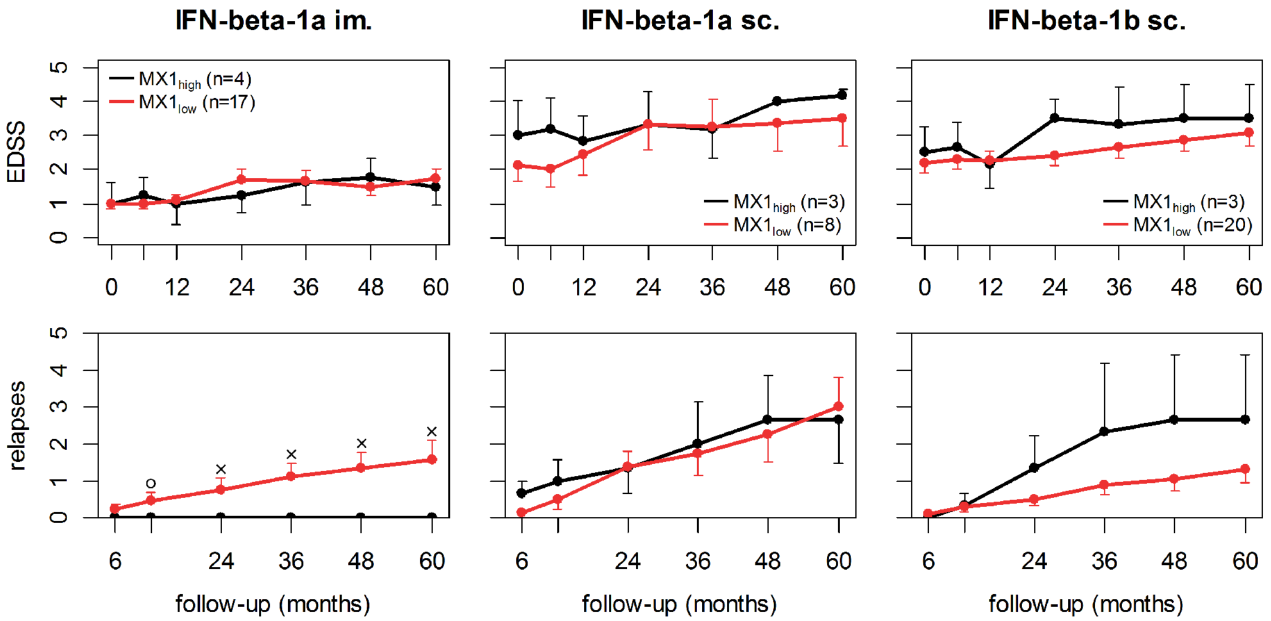

Additional file 7: Cumulative number of relapses and EDSS-defined change of disability displayed for both patient groups and the three different IFN-beta drug preparations.

The change of EDSS over a 5-year follow-up period did not differ significantly between the two cohorts $MX1_{high}$ and $MX1_{low}$ for any type of IFN-beta. However, the EDSS at study onset was lower in the patient group receiving IFN-beta im. (mean EDSS at baseline: 1.0) compared to the patients treated with IFN-beta sc. (mean EDSS at baseline: 2.3). Therefore, patients treated with IFN-beta im. had a lower disease activity at the start of treatment, and they were also more stable in the follow up. Concerning the relapse rates, an analysis of variances (linear model, type II sums of squares and F-test statistic) revealed a significant interaction effect between the two patient groups and the three drug preparations (p-value=0.0002). Of the patients treated with IFN-beta im., no $MX1_{high}$ patient experienced a relapse during follow-up whereas $MX1_{low}$ patients had on average 1.6 relapses. To the contrary, IFN-beta-1b sc. treated patients showed less relapses in the $MX1_{low}$ group than in the $MX1_{high}$ group. However, since the patient groups in our analysis were small, the putative therapy-dependent effect of the endogenous IFN-like activity on long-term disease progression deserves further validation in larger cohorts. ^{o} $p<0.10$, ^{x} $p<0.05$ by Welch's t-test.
