# Supplementary material for: Elevated type I interferon-like activity in a subset of multiple sclerosis patients: molecular basis and clinical relevance
Source: J Neuroinflammation. 2012 Jun 22;9:140. doi: 10.1186/1742-2094-9-140 (PMC3464734; doi:10.1186/1742-2094-9-140)
Supplement: Additional file 8 — The proportion of relapse-free patients during 5 years of follow-up for both patient groups, dependent on the administered IFN-beta drug preparation. [file 1742-2094-9-140-S8.pdf]

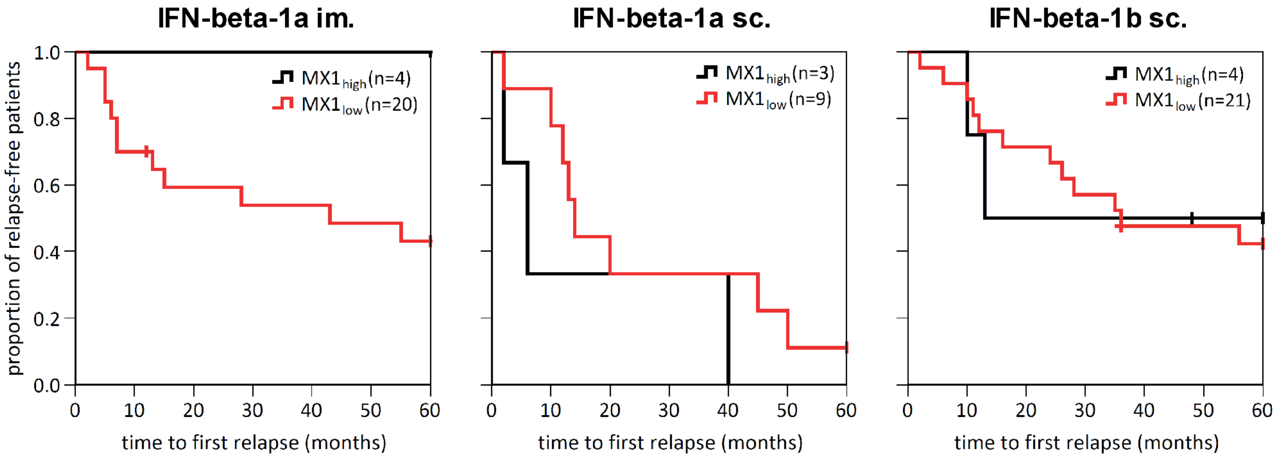

Additional file 8: Relapse-free survival curves of $MX1_{high}$ patients and $MX1_{low}$ patients under therapy with three different IFN-beta drug preparations.

The Kaplan-Meier curves of $MX1_{high}$ patients (black) and $MX1_{low}$ patients (red) have been compared by calculating the logrank test. Right censoring was applied for patients dropping out of clinical surveillance (vertical dashes, n=3). For patients treated with sc. administered IFN-beta the time to a first new relapse after start of therapy was similar between the two patient cohorts, whereas for patients receiving IFN-beta-1a im. the survival curves differed, although this difference was not strictly significant (p-value=0.072).
