# Supplementary material for: Elevated type I interferon-like activity in a subset of multiple sclerosis patients: molecular basis and clinical relevance
Source: J Neuroinflammation. 2012 Jun 22;9:140. doi: 10.1186/1742-2094-9-140 (PMC3464734; doi:10.1186/1742-2094-9-140)
Supplement: Additional file 4 — IFN-beta-related signaling pathways displaying the pharmacological effects on gene expression for the MX1highpatient group after 1 month of treatment with IFN-beta sc. [file 1742-2094-9-140-S4.pdf]

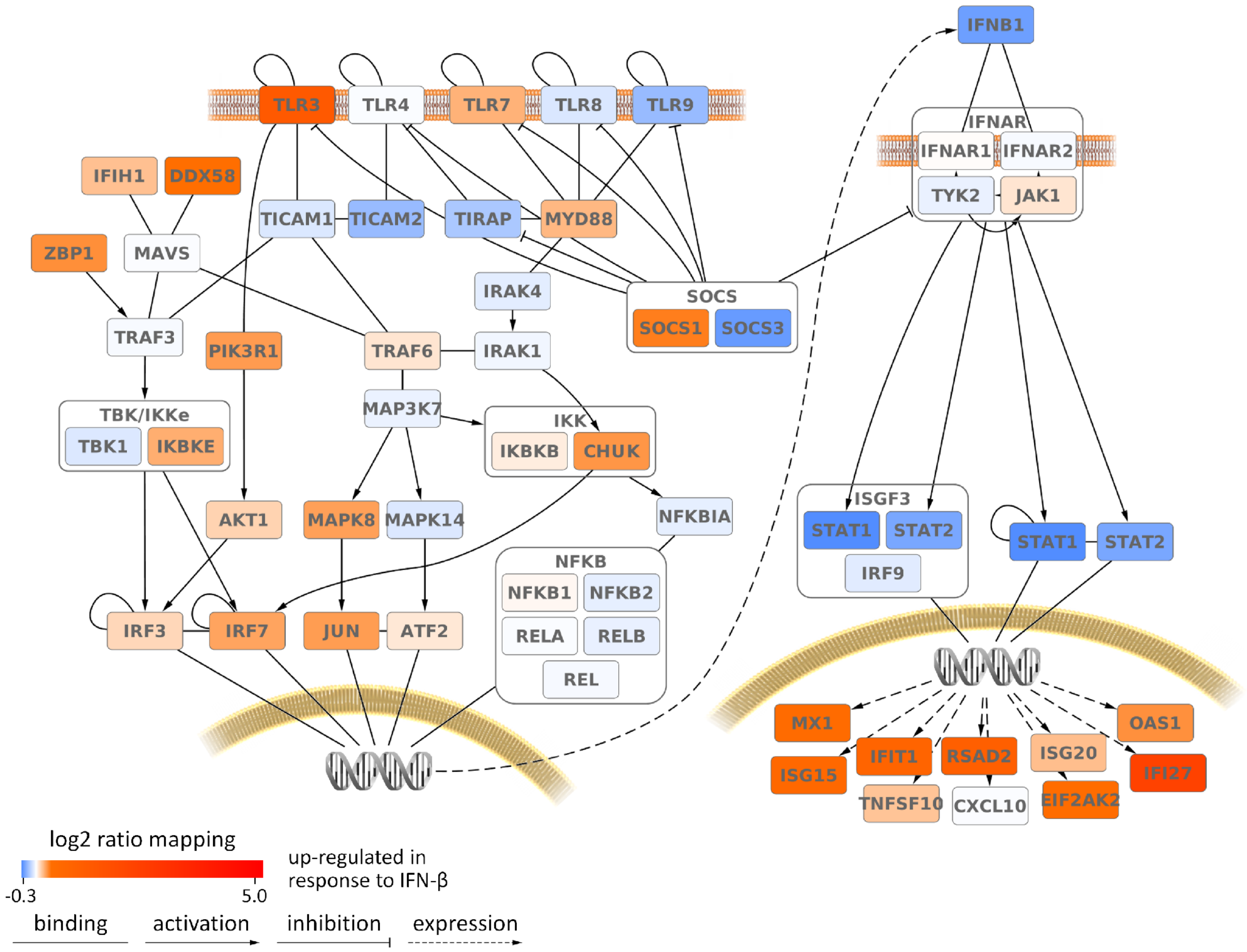

Additional file 4: Gene expression modulation of type I IFN pathway genes in the MX1$_{high}$ group (n=7) during the first month of IFN-beta sc. therapy.

The color of the genes visualizes the log2 ratio of the mean expression levels at one month into therapy versus the mean pre-treatment levels. None of the genes was significantly altered in expression in the MX1$_{high}$ patient group (p-values >0.01). Though not significant, ISGs like RSAD2 (p-value=0.078) and pathogen recognition receptors like TLR3 (p-value=0.016) were slightly up-regulated whilst SOCS3 (p-value=0.047) and STAT1 (p-value=0.375) tended to be down-regulated compared to baseline.
